# Identification of hub genes regulating isoflavone accumulation in soybean seeds *via* GWAS and WGCNA approaches

**DOI:** 10.3389/fpls.2023.1120498

**Published:** 2023-02-14

**Authors:** Muhammad Azam, Shengrui Zhang, Jing Li, Muhammad Ahsan, Kwadwo Gyapong Agyenim-Boateng, Jie Qi, Yue Feng, Yitian Liu, Bin Li, Lijuan Qiu, Junming Sun

**Affiliations:** ^1^ The National Engineering Research Center of Crop Molecular Breeding, Institute of Crop Sciences, Chinese Academy of Agricultural Sciences, Beijing, China; ^2^ Ministry of Agriculture and Rural Affairs (MARA) Key Laboratory of Soybean Biology (Beijing), Institute of Crop Sciences, Chinese Academy of Agricultural Sciences, Beijing, China; ^3^ The National Key Facility for Crop Gene Resources and Genetic Improvement (NFCRI)/Key Laboratory of Germplasm and Biotechnology Ministry of Agriculture and Rural Affairs (MARA), Institute of Crop Sciences, Chinese Academy of Agricultural Sciences, Beijing, China

**Keywords:** soybean, isoflavone, genome-wide association study (GWAS), WGCNA, RNA-Seq

## Abstract

**Introduction:**

Isoflavones are the secondary metabolites synthesized by the phenylpropanoid biosynthesis pathway in soybean that benefits human and plant health.

**Methods:**

In this study, we have profiled seed isoflavone content by HPLC in 1551 soybean accessions grown in Beijing and Hainan for two consecutive years (2017 and 2018) and in Anhui for one year (2017).

**Results:**

A broad range of phenotypic variations was observed for individual and total isoflavone (TIF) content. The TIF content ranged from 677.25 to 5823.29 µg g^-1^ in the soybean natural population. Using a genome-wide association study (GWAS) based on 6,149,599 single nucleotide polymorphisms (SNPs), we identified 11,704 SNPs significantly associated with isoflavone contents; 75% of them were located within previously reported QTL regions for isoflavone. Two significant regions on chromosomes 5 and 11 were associated with TIF and malonylglycitin across more than 3 environments. Furthermore, the WGCNA identified eight key modules: black, blue, brown, green, magenta, pink, purple, and turquoise. Of the eight co-expressed modules, brown (*r* = 0.68***), magenta (*r* = 0.64***), and green (*r* = 0.51**) showed a significant positive association with TIF, as well as with individual isoflavone contents. By combining the gene significance, functional annotation, and enrichment analysis information, four hub genes *Glyma.11G108100*, *Glyma.11G107100*, *Glyma.11G106900*, and *Glyma.11G109100* encoding, basic-leucine zipper (bZIP) transcription factor, MYB4 transcription factor, early responsive to dehydration, and PLATZ transcription factor respectively were identified in brown and green modules. The allelic variation in *Glyma.11G108100* significantly influenced individual and TIF accumulation.

**Discussion:**

The present study demonstrated that the GWAS approach, combined with WGCNA, could efficiently identify isoflavone candidate genes in the natural soybean population.

## 1 Introduction

Soybean isoflavones are of great importance because of their positive impact on human health, including the treatment and prevention of various types of cancers (prostate cancer, breast cancer etc.) (Nielsen and Williamson, 2007; Phetnoo et al., 2013), cardiovascular disease, osteoporosis, and metabolic syndrome (Cai et al., 2004; Mozaffarian et al., 2011; Bradbury et al., 2014). In plants, isoflavones can resist adverse stress and promote the growth and reproduction of rhizobia, root nodule development, and nitrogen fixation (Sugiyama et al., 2017; Darwish et al., 2022; Wang et al., 2022). Soybean seed isoflavones contain 12 components which are divided into four groups, daidzein, genistein, glycitein (aglycones), daidzin, glycitin, genistin (glycosides), acetyldaidzin, acetylglycitin, and acetylgenistin (acetylglycosides), and malonyldaidzin, malonylglycitin, malonylgenistin (malonylglycosides) (Kim et al., 2014; Azam et al., 2021). The malonyldaidzin, malonylglycitin, and malonylgenistin are the most abundant form of the isoflavones, while aglycones are present in very small amounts but have higher phytoestrogenic activity and more bioavailability in humans (Nielsen and Williamson, 2007; Park et al., 2016; Azam et al., 2020). Improving soybean isoflavone content through conventional breeding and metabolic engineering is a complementary way for the biofortification of food crops to combat isoflavone deficiency (De Steur et al., 2014).

Isoflavone content is controlled by multiple genes, and there are often complex interaction mechanisms among various enzyme genes in its synthesis path, which jointly determine isoflavone biosynthesis. The metabolic pathway controlling the synthesis of soybean isoflavones in plants is very complex (Wang and Murphy, 1994; Bennett et al., 2004). The synthesis of soybean isoflavones starts from the synthesis of phenylpropionic acid. The original substrate of isoflavones is phenylalanine, which is catalyzed by phenylalanine lyase (PAL), cinnamate-4-hydroxylase (C4H), and 4-coumarin coenzyme A ligase (4CL), respectively to produce p-coumaroyl COA, Isoliquiritigenin chalcone and chalcone were formed with malonyl COA of 3 molecules under the co catalysis of chalcone synthase (CHS) and chalcone reductase (CHR). Isoliquiritigenin chalcone is catalyzed by chalcone isomerase (CHI) to produce liquiritigenins (Ralston et al., 2005), which are then catalyzed by isoflavone synthase genes (*IFS1 and IFS2*) to their corresponding isoflavones (Akashi et al., 1999; Jung et al., 2000; Dhaubhadel et al., 2003). Among isoflavone synthase genes, *IFS2* has a higher expression level in the embryo and seed pods, while *IFS1* has higher expression in roots and seed coats. In addition, various kind of MYB transcription factors (CCA1, R2R3, and R1) helps in isoflavone accumulation by regulating the isoflavone synthesis genes related to phenylpropanoid biosynthesis pathways (Bian et al., 2018; Sarkar et al., 2019). The R2R3-MYB transcription factor *GmMYB29*, *GmMYB102*, *GmMYB280*, *MYB502*, *GmMYB100* regulate isoflavone accumulation by activating the *IFS1*, *IFS2* and *CHS8* enzymes (Yan et al., 2015; Sarkar et al., 2019). The CCA1-like R1 MYB transcription factor *GmMYB133* regulates isoflavone biosynthesis by activating the promoters of *CHS8* and *IFS2* (Bian et al., 2018). A dual-function C2H2 zinc-finger transcription factor *GmZFP7* has recently been shown to divert metabolic flow to isoflavone by increasing the expression of *GmC4H*, *Gm4CL*, *GmCHS*, *GmCHR*, and *GmIFS2* while decreasing the expression of *GmF3H1* in soybean seeds. (Feng et al., 2023).

Soybean isoflavones are quantitative traits regulated by multiple genes. The genotyping by sequencing (GBS) approach and SNP genotyping have substantially expanded the application of GWAS to soybeans (Lee et al., 2015; Sonah et al., 2015; Torkamaneh and Belzile, 2015). Natural population based GWAS have more recombination events than biparental populations, resulting in less short LD regions and higher precision and accuracy of marker phenotype association (Duan et al., 2022; Liang et al., 2022). These approaches have been utilized in GWAS to identify genomic regions associated with resistance to biotic and abiotic stress, including soybean cyst nematode, abiotic stress, seed quality traits such as oil and protein content, and yield related traits (Hwang et al., 2014; Cao et al., 2017; Zeng et al., 2017; Zhao et al., 2017). Furthermore, weighted gene co-expression network (WGCNA) analysis is a powerful tool for describing gene expression correlations using microarray or RNA-seq data. The WGCNA is an effective method to narrow down the range of candidate genes (Schaefer et al., 2018). Recently, GWAS combined with WGCNA has been applied to identify the genes responsible for salt tolerance in maize, silique length in *Brassica napus*, and root growth dynamics in rapeseed (Li et al., 2021; Ma et al., 2021; Wang et al., 2021). However, no study has used the GWAS and the WGCNA to explain the gene networks and molecular regulatory mechanisms that govern isoflavone regulation in soybean. Therefore, the present study aimed to identify the genomic regions and candidate genes involved in the isoflavone biosynthesis pathway using GWAS coupled with WGCNA in 1551 soybean accessions.

## 2 Research materials and methods

### 2.1 Planting materials

A total of 1551 natural population panel of diverse soybean accessions was used in this study. The accessions were selected from a mini core collection developed by Qiu et al. (2009) based on their availability at the soybean genetic resource research group of the Institute of Crop Sciences, Chinese Academy of Agricultural Sciences (CAAS). The origin and number of soybean accessions from each country are Brazil (8), Canada (6), China (1283), Colombia (1), East Europe (3), Germany (4), Italy (2), Japan (21), Nigeria (1), North Korea (1), Russia (22), South Korea (4), Thailand (1), USA (194). Information on each accession is also presented in **Supplementary Table 1**. Field trials were conducted at three locations (Changping, Beijing (40^°^ 13′ N and 116^°^ 12′ E), Sanya, Hainan (18^°^ 24′ N and 109^°^ 5′ E) in 2017 and 2018, while, for only 2017, planted in Hefei, Anhui (33°61′ N and 117 °E). A randomized incomplete block design was employed to sow the cultivars, with the various planting sites serving as replications. The cultivars were replicated across different sites due to a large number of cultivars and the scarcity of available land resources. Each cultivar’s seeds were sown in 3 m long rows with 0.5 m inter-row and 0.1 m intra-row spacing. Fertilizer containing 30 kg/ha, 40 kg/ha, and 60 kg/ha of nitrogen, phosphorous, and potassium was applied to the field, respectively. From planting until harvest, the advised agronomic procedures were used. The seeds from each accession were pooled and used for soybean seed isoflavone determination (Azam et al., 2020; Azam et al., 2021).

### 2.2 Extraction and quantification of isoflavones

The isoflavone contents were determined using a previously reported method (Sun et al., 2011) and as follows. Around 20 g seeds of each accession were grounded by a cyclone mill (IKA, A10 basic, Rheinische, Germany). Approximately 0.1 g of the finely ground powder was placed in a 10 mL tube pre-filled with 5 mL of a solution containing 0.1% (v/v) acetic acid and 70% (v/v) ethanol and shaken for 12 hours on a twist mixer (TM – 300, AS ONE, Osaka, Japan). The mixture was centrifuged for 10 min at 6000 rpm, and the supernatant was filtered using a 0.2 μm YMC Duo filter (YMC Co., Kyoto, Japan). Samples were stored at 4°C prior to use and measured for isoflavones using an Agilent HPLC system (Agilent 1260, Santa Clara, CA, USA) having YMC ODS AM-303 column (250 mm × 4.6 mm I.D., S-5 μm, 120 Å, YMC Co., Kyoto, Japan). The identification and quantification of the isoflavone contents were carried out using the following isoflavone standards: daidzein (DE), glycitein (GLE), genistein (GE), daidzin (D), glycitin (GL), genistin (G), malonyldaidzin (MD), malonylglycitin (MGL), malonylgenistin (MG), acetyldaidzin (AD), acetylglycitin (AGL), and acetylgenistin (AG). The detected isoflavone component concentrations were determined using the formula provided by (Sun et al., 2011).

### 2.3 Association analysis and candidate gene prediction and annotation

A total number of 6,149,599 SNPs with MAF 0.01 from previously sequenced 2,241 soybean accessions were used for GWAS analysis (Li et al., 2022). GWAS was performed using the compressed mixed linear model (cMLM) in the GAPIT program (Lipka et al., 2012), where the first three principal component analysis (PCA) values were included as fixed effects in the mixed model to correct for stratification. The threshold for significance was estimated to be approximately *P* = 1 × 10^-6^ (that is, 1/6,149,599) by the Bonferroni correction method. These 6,149,599 SNPs were distributed equally across the 20 soybean chromosomes (one SNP per 154.3 bp). The extent of model fitting was confirmed using a quantile-quantile (Q-Q) plot for the expected and obtained *p*-values of each SNP to evaluate how much a significant result was produced by the analysis than expected by chance. The Manhattan plots for the isoflavone contents for each of the five environments were generated from GAPIT (Lipka et al., 2012). The Phytozome database (http://www.phytozome.org/) and the SoyBase database (http://www.soybase.org/) were used to predict and annotate the candidate genes.

### 2.4 RNA seq-analysis

The four soybean varieties Luheidou (LHD), Zhonghuang 13 (ZH13), Zhonghuang 35 (ZH35), and Nanhuizao (NHZ), varying in their isoflavone contents, were used as materials for RNA seq-analysis. About 20 seeds were harvested at different developmental stages (R5 to R8) after 7 days intervals. Each sample was set with three replications for isoflavone contents, and RNA extraction. The total RNAs were extracted using the TRIzol method. The high-quality RNA samples were sent for RNA-seq analysis to BLgene co. LTD (Beijing, China). HISAT2 was used to map the clean RNA-seq data onto the reference genome (Kim et al., 2015). FeatureCounts calculated the transcriptional abundance and gene expression count matrix (Liao et al., 2014). TPM (transcripts per million) was used as the expression level, and log10 (TPM + 1) was used to standardize it (Feng et al., 2023).

### 2.5 Weighted gene co-expression network analysis

The transcriptome data of (LHD, NHZ, ZH13, and ZH35) at different seed developmental stages was used for the WGCNA. The R WGCNA (v1.47) package was used to create the weighted gene co-expression network (Langfelder and Horvath, 2008). The gene expression values were imported into WGCNA to construct co-expression modules using the automatic network construction with default settings. The phenotype data was imported into the WGCNA package, and correlation-based connections between phenotypes and gene modules were computed using the default settings. Pearson’s correlation between all gene pairs was first determined to create a matrix of adjacencies. Using the TOM similarity function, this matrix was transformed into a Topological Overlap Matrix (TOM) (Zhang and Horvath, 2005). Finally, modules on the dendrogram were discovered using the R package dynamicTreeCut method (Langfelder et al., 2008). The hub genes are usually characterized by high gene significance (GS, association between gene expression and traits) and module membership (MM, correlation between gene expression and module eigengene) values.

### 2.6 Gene ontology analysis

The GO enrichment analysis was performed to identify GO categories based on the SoyBase database (http://soybase.org/) and detect those over/under-represented. The significant enriched GO terms (*P* < 0.05) for biological processes, the cellular process, and molecular processes were further identified using PlantRegMap online tool (http://plantregmap.cbi.pku.edu.cn/go_result.php) and were visualized REVIGO (http://revigo.irb.hr/) (Supek et al., 2011).

## 3 Results

### 3.1 Variations among seed isoflavone contents in soybean natural population

**Figure 1:**
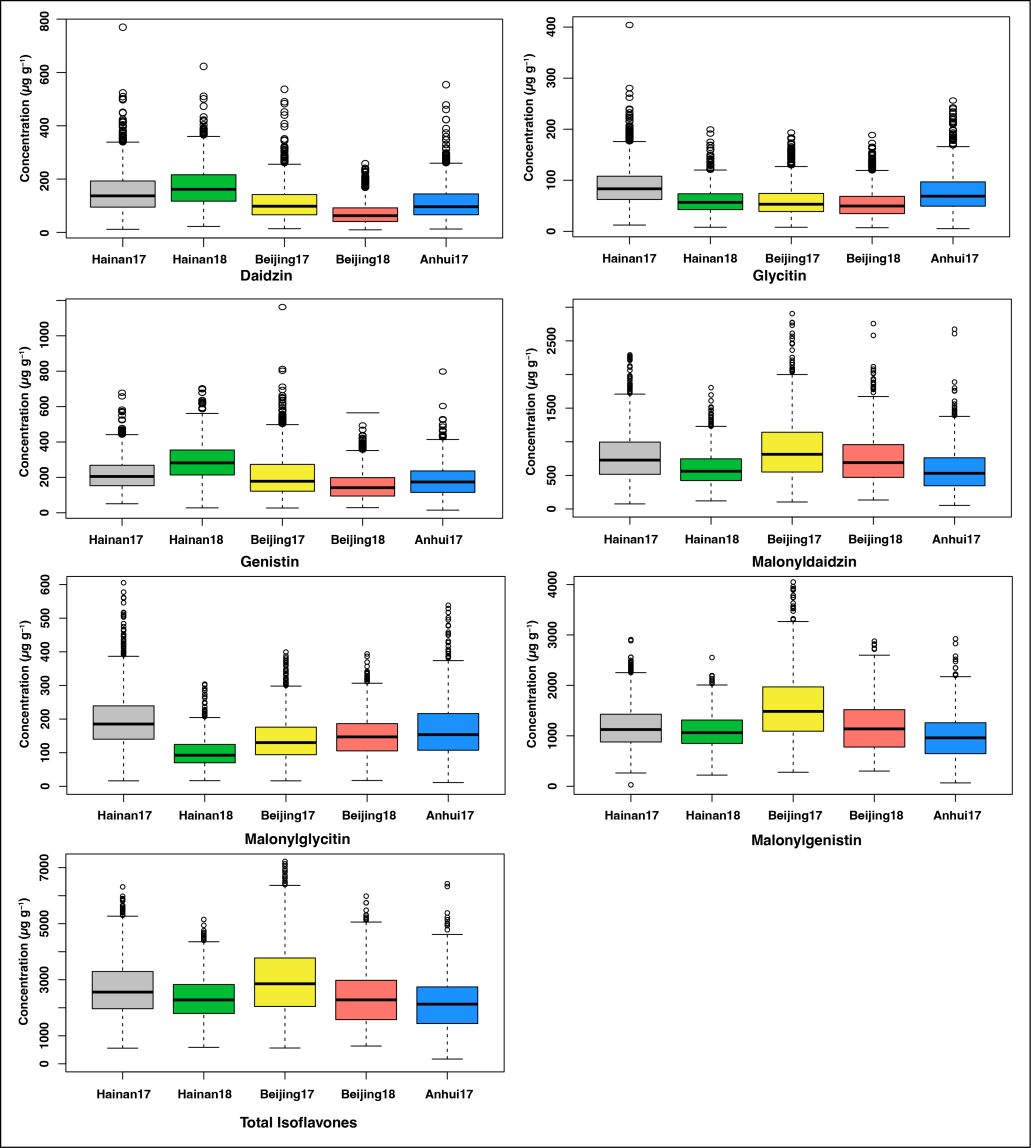
Individual and TIF content in five environments (Hainan 2017 Hainan 2018, Beijing 2017, Beijing 2018, and Anhui 2017).

The individual and TIF content was profiled in soybean accessions collected from distinct regions of China and other countries that have grown across three locations over two years. The mean TIF content of the 1551 soybean natural population grown across five environments is presented in **Supplementary Table 1**. The mean TIF content of the soybean accessions ranged from 677.25 to 5823.29 µg g^-1^ (Azam et al., 2020; Azam et al., 2021). The individual and TIF content of the soybean accessions in five environments are presented in **Figure 1**. The correlations among the five environments for individual and TIF content are presented in **Supplementary Figure 1**. The higher levels of daidzin (172.7 µg g^-1^), genistin (290 µg g^-1^) were observed in Hainan 2018, followed by Hainan 2017 (daidzin (152.4 µg g^-1^, genistin (218.8 µg g^-1^). The higher levels of malonyldaidzin (888.3 µg g^-1^), malonylgenistin (1574.1µg g^-1^), and TIF (3012.3 µg g^-1^) were observed in Beijing 2017, followed by Hainan 2017 (malonyldaidzin (789.9 µg g^-1^), malonylgenistin (1183.1 µg g^-1^) and TIF (2685.5 µg g^-1^), while Anhui 2017 showed lower levels of these components (malonyldaidzin (589.2 µg g^-1^), malonylgenistin (984.1 µg g^-1^) and TIF (2153.1 µg g^-1^). While higher levels of malonylglycitin (208.2 µg g^-1^) were observed in Hainan 2017, followed by Anhui 2017 (168.2 µg g^-1^) and lowest in Hainan 2018 (100.1 µg g^-1^) (**Figure 1**).

**Figure 2:**
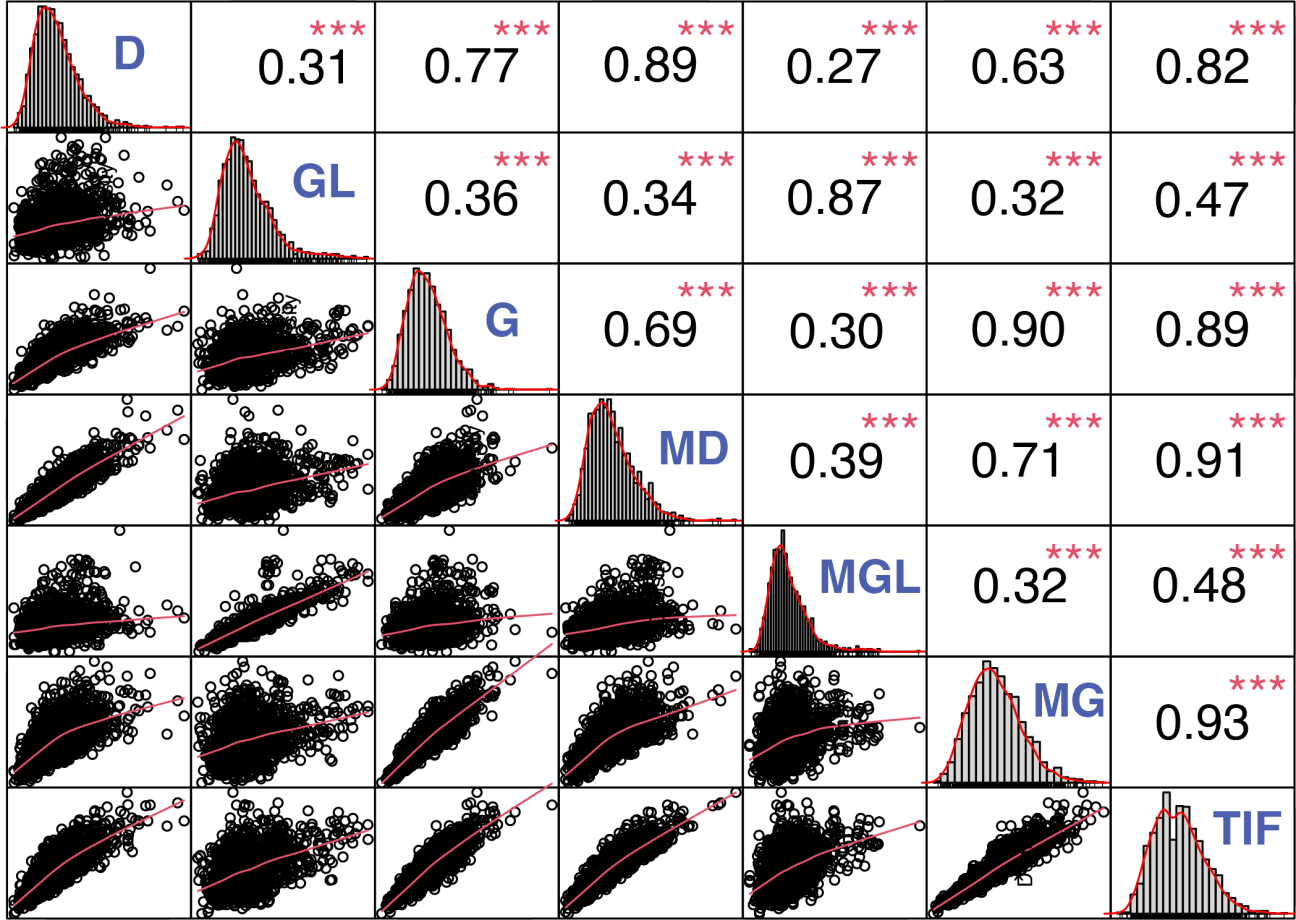
Correlation analysis among the individual and TIF content in soybean seeds. *, **, and *** represent significance at *p* < 0.05, 0.01, and 0.001, respectively. D, Daidzin; GL, Glycitin; G, Genistin; MD, Malonyldaidzin; MGL, Malonylglycitin; MG, Malonylgenistin; TIF, Total isoflavone.

Furthermore, Pearson’s correlation was performed to reveal the association between individual and TIF content. TIF content was positively associated with individual isoflavone contents (**Figure 2**). Malonylgenistin, Malonyldaidzin, genistin, and daidzin showed the highest correlation with TIF content (*r* = 0.93***, *r* = 0.91***, *r* = 0.89***, *r* = 0.82***, respectively), followed by malonylglycitin and glycitin (*r* = 0.48***, *r* = 0.47***, respectively). Furthermore, glycosides showed highly significant positive correlations with their respective malonylglycosides, genistin and malonylgenistin (*r* = 0.90***), daidzin and malonyldaidzin (*r* = 0.89***), and glycitin and malonylglycitin (*r* = 0.87***) (**Figure 2**).

### 3.2 GWAS reveals candidate loci underlying seed isoflavone contents

**Figure 3:**
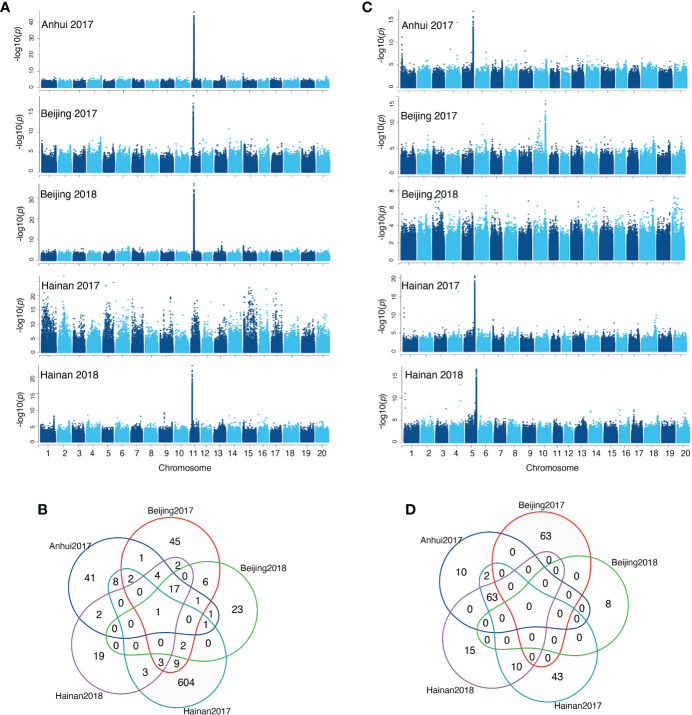
**(A)** Manhattan plots of malonylglycitin for five environments, **(B)** Venn plot for malonylglycitin genes in five environments, **(C)** Manhattan plots of TIF content for five environments, **(D)** Venn plot for TIF content genes in five environments.

The phenotypic and genotypic data for 1551 diverse soybean accessions were used for GWAS analysis to identify putative loci associated with isoflavone contents in the individual environment (Hainan 2017, Hainan 2018, Beijing 2017, Beijing 2018, and Anhui 2017). The principal component analysis (PCA) was used for scanning the population stratification. The landrace group overlapped partially with the improved cultivar group, indicating a broad genetic variation within this set of 1551 soybean accessions. Meanwhile, clear clustering based on planting region was observed; the first two PCs accounted for 40.47% of the genetic variation, demonstrating that the first two PCs uncommonly affect the mapping population. The average distance over which LD decays to half of its maximum value in soybean was 97kb (**Supplementary Figures 2A, B**) GWAS identified 11704 genome-wide distributed SNPs that were significantly (-log10P>6) associated with isoflavone levels with P-values ​​ranging from 9.99e-07 to 7.30e-30, the detailed information is listed in **Supplementary Table 2**. Of the 11704 significant SNPs, 53.8% were annotated in intergenic regions, 19.9% in the upstream and downstream regions, 14% in the intron regions. Herein, 8786 SNPs (75%) identified from the GWAS were located within the regions of previously reported QTLs for isoflavone in soybean. In total, 2,018 known genes were mapped by the significant SNPs, which include 29 isoflavone biosynthesis enzymes and 18 MYB transcription factors; of these, 417, 261, 316, 428, 307, 847, and 230 genes were significantly associated with daidzin, glycitin, genistin, malonyldaidzin, malonylgenistin, malonylglycitin, and TIF content, respectively (**Supplementary Tables 2**, **3**). Interestingly, a significant region (8147595 to 8315102bp) has been identified on chromosome 11 across four environments associated with malonylglycitin and contains 18 genes (**Figures 3A, B**), including eight enzymes and three transcription factors MYB (1), bZIP (1) and zinc finger (1). Furthermore, a significant region on Chromosome 5 related to TIF content across three environments spanning from 41760764 to 42234431 bp encoded 63 candidate genes (**Figures 3C, D**), including seven key enzymes, and four transcription factors WD40 (1), bZIP (1) and zinc finger (2) (**Supplementary Tables 4**, **5**).

### 3.3 Identification of key modules possessing candidate genes *via* WGCNA

**Figure 4:**
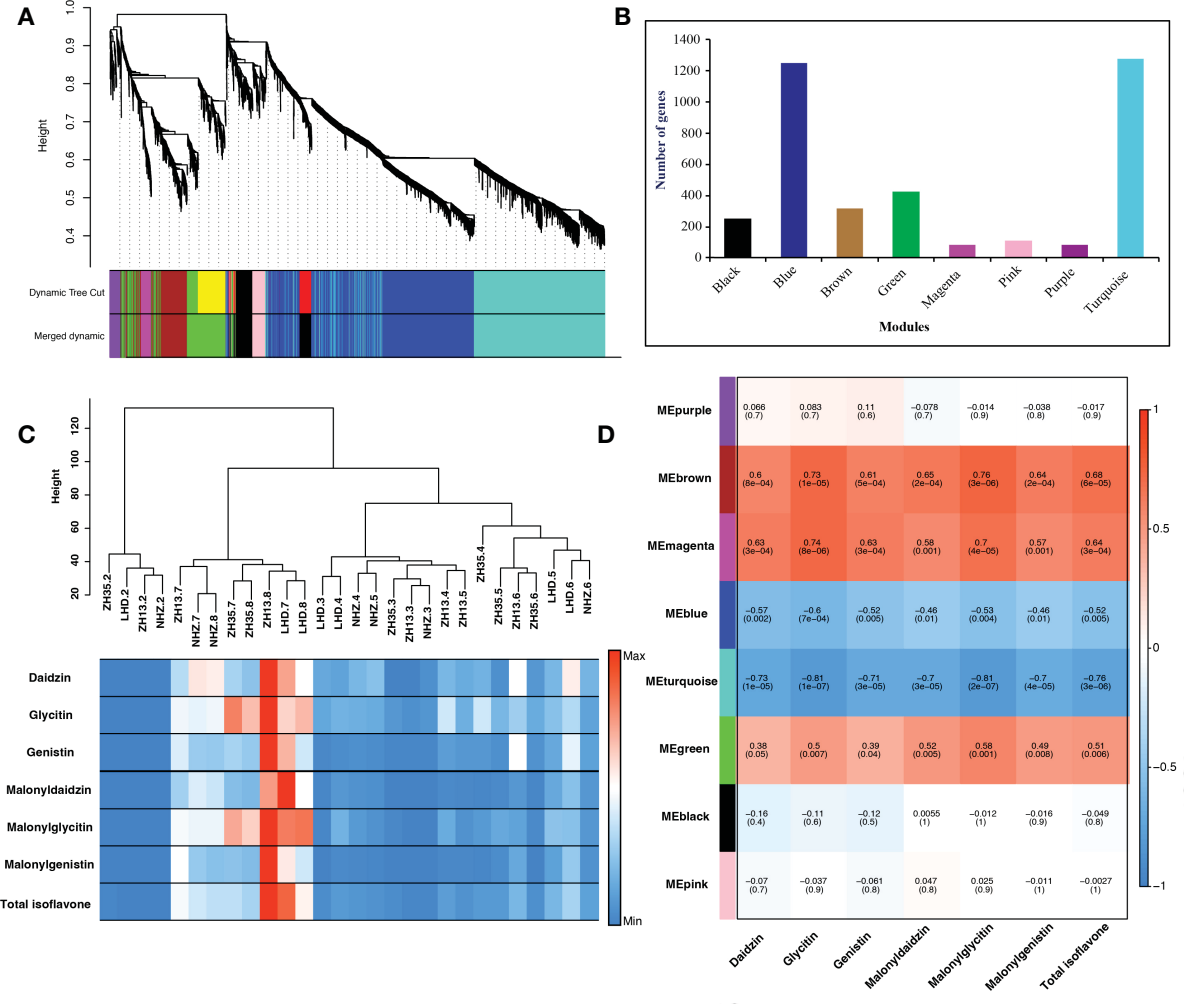
**(A)** Module clustering, different colors represent different modules. **(B)** Number of genes in each module. **(C)** Sample dendrogram and trait heatmap, each row corresponds to the isoflavone content, while each column corresponds to seed samples of four soybean cultivars (LHD, NHZ, ZH13, and ZH35) collected at different seed developmental stages (R5-R8). The right panel represents the minimum (blue color) and maximum (red color) isoflavones accumulation at different seed developmental stages. **(D)** Module trait relationship, each row corresponds to a module, while each column corresponds to the isoflavone content. The left panel shows the modules, while the right panel shows positive (red, 1) and negative (blue, − 1) correlations.

The transcriptome data of different seed developmental stages were used for WGCNA, which provided new genomic insights to better understand the molecular mechanisms underlying isoflavone accumulation in soybean seed. The candidate genes identified in the linkage disequilibrium regions obtained through GWAS analysis were blast searched against the transcriptome data of soybean cultivars collected at different seed developmental stages (R5-R8) to identify common genes for WGCNA analysis. The WGCNA identified eight key modules, namely, black, blue, brown, green, magenta, pink, purple, and turquoise, possessing 253, 1251, 316, 426, 82, 113, 83, and 1275 genes, respectively (**Figures 4A, B**).

**Figure 5:**
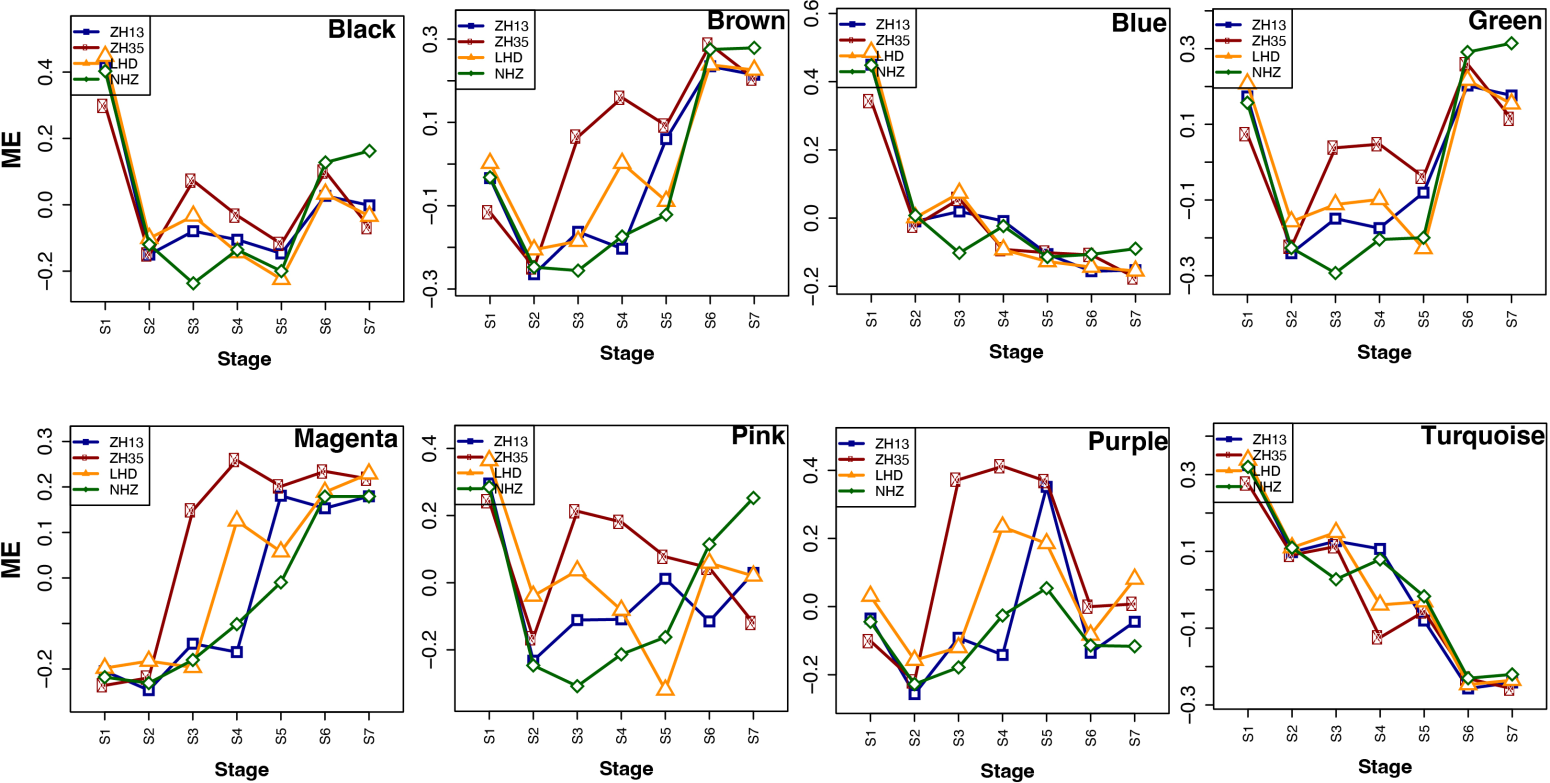
Expression profiles of the modules at different seed developmental stages in four soybean cultivars.

**Figure 6:**
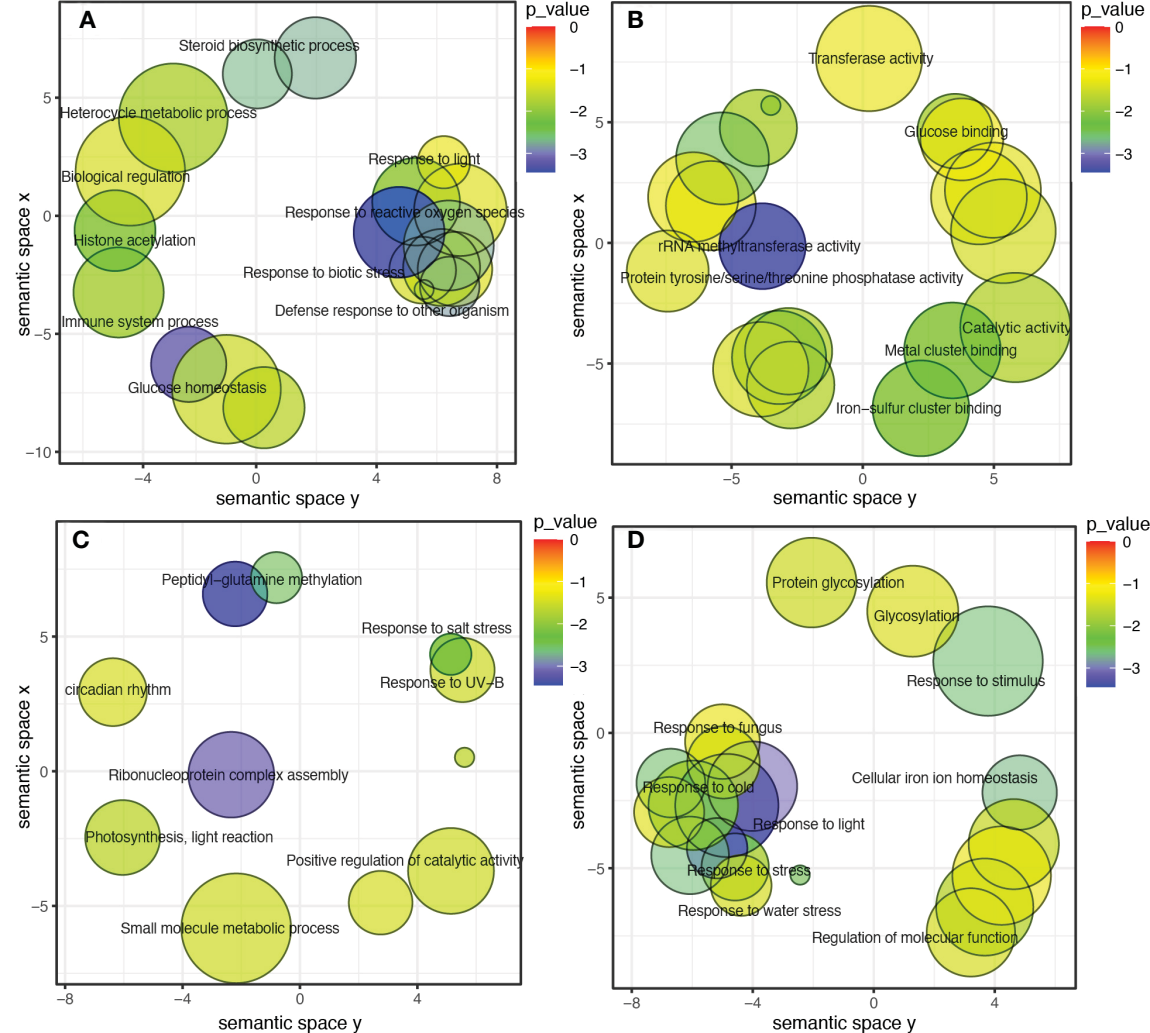
**(A)** GO categories for biological process, brown module. **(B)** GO categories for molecular function, brown module. **(C)** Categories for biological process, green module. **(D)** Categories for biological process, magenta module.

To further investigate the modules containing genes involved in isoflavone synthesis, Pearson’s correlation analysis was performed. Of the eight co-expressed modules, brown (*r* = 0.68***), magenta (*r* = 0.64***), and green (*r* = 0.51**) showed significant positive correlations with TIF, as well as with individual isoflavone contents. The sample dendrogram and trait heat map also revealed that the isoflavone accumulation is higher at late seed developmental stages (**Figures 4C, D**). Furthermore, genes in brown, magenta, and green modules showed higher expression patterns at late seed developmental stages. It is already established that higher isoflavone accumulations were observed in the soybean seeds at later developmental stages (**Figure 5**). To further investigate the relationship of genes in each of the positive modules with isoflavone synthesis, the correlation between gene significance (GS)and module membership (MM) was carried out. Out of 8 modules, the brown module showed a highly positive correlation with TIF (*r* = 0.71***), followed by magenta (*r* = 0.7***), while the lowest was observed in the green module (*r* = 0.44***) (**Supplementary Figure 3**). Furthermore, the GO enrichment analysis revealed that the brown module possesses genes linked to defense response to bacterium (GO:0042742), defense response to other organism (GO:0098542), defense response, incompatible interaction (GO:0009814), response to reactive oxygen species (GO:0000302). Similarly, genes present in the magenta module are response to stress (GO:0006950), response to water deprivation (GO:0009414), cellular response to red or far-red light (GO:0071489) enzyme regulator activity (GO:0030234), and genes in the green module are involved in the regulation of circadian rhythm (GO:0042752), response to UV (GO:0009411), response to salt stress (GO:0009651) are engaged in biotic and abiotic stresses (**Figures 6A-D**). Current results suggest that genes present in the above-mentioned modules, i.e., brown, magenta, and green, might be involved in isoflavone accumulation in soybean seeds; they can play important roles in the isoflavone synthesis pathway.

Further, gene annotation and gene significance information were used to identify hub genes in brown, magenta, and green modules. Based on the gene significance and annotation information, 27 key candidate genes were identified and are presented in **Table 1**. These candidate genes include 9 transcription factors (4 MYB, 3 WD40, 1 WRKY, and 1 Zinc finger) and 12 key enzymes, including glucosyl transferases, isoflavone 2’-hydroxylase, etc. Two MYB transcription factors in the brown module, *MYB133* (*Glyma.07G066100*) and *MYB121* (*Glyma.15G176000*) were identified as positive regulators of isoflavone biosynthesis from previous studies. While in the magenta module, we identified a cytochrome P450 enzyme, isoflavone 2’-hydroxylase (*Glyma.16G149300*), a positive regulator of isoflavones. Interestingly, four hub genes *Glyma.11G108100*, *Glyma.11G107100*, *Glyma.11G106900*, and *Glyma.11G109100* encoding, basic-leucine zipper (bZIP) transcription factor, MYB4 transcription factor, early responsive to dehydration, and PLATZ transcription factor, respectively were identified in brown and green modules. These four hub (*Glyma.11G108100*, *Glyma.11G107100*, *Glyma.11G106900*, and *Glyma.11G109100*) genes were also present in the candidate region located on Chromosome 11 identified by GWAS and matched with previously identified QTLs. Isoflavones play an important role in biotic and abiotic stress in plants, and MYB transcription factors help in isoflavone accumulation by regulating key isoflavone synthase genes (*IFS1* and *IFS2*). Therefore, the identified transcription factors (bZIP, MYB, PLATZ) might be involved in the isoflavone accumulation as they are also helping plants to adapt to various kinds of biotic and abiotic stresses.

**Table 1:** List of candidate genes for individual and TIF content in brown, magenta, and green modules.

Gene ID	Module	GS.TIF	*p*.GS.TIF	Annotation
*Glyma.11G108100*	Brown	0.77	1.73E-08	Basic-leucine zipper (bZIP) transcription factor
*Glyma.17G085800*	Brown	0.76	2.02E-06	S-adenosyl-L-methionine methyltransferase
*Glyma.07G100700*	Brown	0.76	2.20E-06	MYB transcription factor
*Glyma.08G125100*	Brown	0.75	3.10E-06	Cytochrome P450
*Glyma.06G094900*	Brown	0.74	5.79E-06	WD40 repeat family protein
*Glyma.11G109100*	Brown	0.74	1.73E-08	PLATZ transcription factor
*Glyma.11G106900*	Brown	0.72	1.26E-05	Early responsive to dehydration
*Glyma.13G069200*	Brown	0.67	0.000121	Zinc finger family protein
*Glyma.14G054400*	Brown	0.66	0.000115	UDP-glucosyl transferase
*Glyma.07G066100*	Brown	0.62	0.000402	MYB transcription factor *MYB133*
*Glyma.18G114800*	Brown	0.61	0.000646	WD40 repeat family protein
*Glyma.15G053400*	Brown	0.61	0.000851	Potassium transporter
*Glyma.08G240800*	Brown	0.59	0.000852	WRKY transcription factor
*Glyma.15G176000*	Brown	0.56	0.001773	MYB transcription factor *MYB121*
*Glyma.03G187700*	Green	0.79	3.37E-07	UDP-glucosyl transferase
*Glyma.15G048600*	Green	0.69	4.01E-05	Mitogen-activated protein kinase
*Glyma.01G092100*	Green	0.64	0.000216	Zinc finger family protein
*Glyma.10G216200*	Green	0.55	0.002163	Heat shock protein
*Glyma.06G171900*	Green	0.53	0.003656	4-coumarate-coa ligase
*Glyma.02G267800*	Green	0.46	0.013048	WD40 repeat protein
*Glyma.05G242800*	Green	0.41	0.034201	ATP-dependent RNA helicase A-like protein
*Glyma.11G107100*	Green	0.44	0.018738	Transcription factor MYB4
*Glyma.04G243600*	Green	0.36	0.041367	MYB transcription factor
*Glyma.17G112400*	Magenta	0.66	0.000119	N-acetylglucosaminyltransferase
*Glyma.14G198600*	Magenta	0.65	0.000161	UDP-Glycosyltransferase
*Glyma.02G263500*	Magenta	0.61	0.000663	S-adenosyl-L-methionine methyltransferases
*Glyma.16G149300*	Magenta	0.42	0.023798	Isoflavone 2’-hydroxylase

**GS.TIF**, gene significance total isoflavone; **p.GS.TIF**, significant level.

### 3.4 Natural variation in *Glyma.11G108100* contributes to isoflavone accumulation

**Figure 7:**
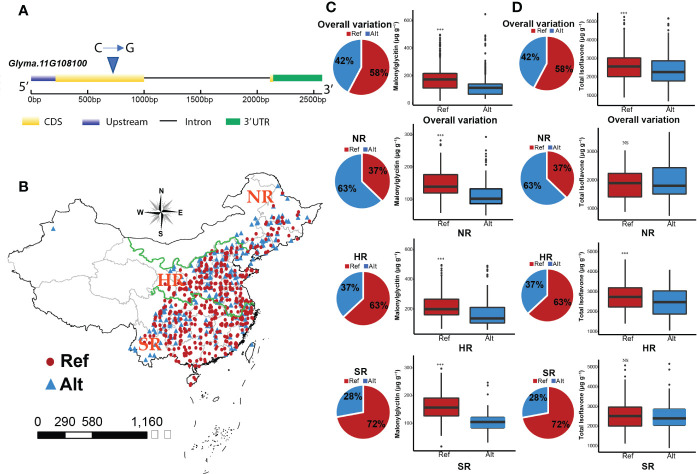
**(A)** Polymorphism that occurred in *Glyma.11G108100*. **(B)** Geographical distribution of *Glyma.11G108100* (NR, Northern region; HR, Huang Huai Hai valley region; SR, Southern region). **(C)** Natural variation of *Glyma.11G108100* for malonylglycitin content. **(D)** Natural variation of *Glyma.11G108100* for TIF content.

Natural variation of *Glyma.11G108100* was identified by using the soybean functional genomics & breeding (SoyFGB v 2.0) database (https://sfgb.rmbreeding.cn/) (Zheng et al., 2022). Based on the phytozome database (https://phytozome-next.jgi.doe.gov), the coding region of *Glyma.11G108100* contains 813 nucleotides, which encodes 270 amino acids with two exons and one intron. The causal SNP was in the exonic region (**Figure 7A**). Williams82 provided the reference allele (C), while the polymorphism that occurred resulted in the alternate allele (G). The geographical distribution of C and G alleles is presented in **Figure 7B**. The overall variation revealed significant differences in malonylglycitin content for C and G alleles which have 58% and 42% distribution in the soybean germplasm. The C allele had higher malonylglycitin content (183.3 µg g^-1^) than the G allele (126.8 µg g^-1^). The regional distribution of these alleles showed significant differences in malonylglycitin content in NR, HR, and SR regions. The distribution of the C allele in NR, HR, and SR regions is 37%, 63%, and 72%, respectively, while the G allele is 63%, 37%, and 28%, respectively. The C allele had higher malonylglycitin content in NR (150.4µg g^-1^), HR (222.1µg g^-1^), and SR (159.7µg g^-1^) compared with the G allele (**Figure 7C**). Furthermore, the natural variation of *Glyma.11G108100* also influenced the TIF content accumulation in soybean seed. The overall variation revealed significant differences in TIF content for C and G alleles, with 58% and 42% distribution in the soybean germplasm. The C allele had higher TIF content (2568.8 µg g^-1^) compared with the G allele (2387.7 µg g^-1^). The regional distribution of these alleles showed significant differences for TIF content in the HR region, while non-significant differences for NR and SR regions. The distribution of the C allele in the HR region is 63%, and the G allele is 37%. The TIF content of the C allele (2793.9 µg g^-1^) was significantly higher than the G allele (2509.5 µg g^-1^) in the HR region (**Figure 7D**). The polymorphism in *Glyma.11G108100* showed significant variations for individual and TIF content across soybean germplasm and regions, suggesting that it might be associated with isoflavone accumulation in soybean.

## 4 Discussion

Soybean isoflavones are of great interest owing to their beneficial impact on plant and human health. Increasing isoflavone concentration in soybean is one of the major goals of soybean breeders; however, the narrow genetic diversity of the soybean germplasm constrains the improvement of the isoflavones (Qiu et al., 2009). In this study, we determined the isoflavone composition from the core germplasm of soybean accessions grown at three locations for two years. Significant differences were observed for individual and TIF content across different environments. The TIF concentration ranged from 677.25 to 5823.29 µg g^-1^ across all the examined environments. Malonylglycosides were identified as major isoflavone contents (Zhang et al., 2014; Azam et al., 2020). Furthermore, glycosides and malonylglycosides showed positive associations as they are synthesized by the action of key isoflavone biosynthesis enzymes (glucosyltransferase and malonyltransferase) *via* common branches in the phenylpropanoid pathway (Yu and Mcgonigle, 2005; Barnes, 2010). The phenotypic variation of individual and TIF content demonstrated significant differences among the soybean accessions, growing environments, and growing years which suggests that genetic as well as environmental factors affect isoflavone accumulation in soybean seeds (Tsai et al., 2007; Rasolohery et al., 2008; Zhang et al., 2014; Pei et al., 2018; Azam et al., 2023).

Isoflavones are typical quantitative traits; many QTLs for individual and TIF content distributed on most soybean chromosomes have been detected in several studies (Akond et al., 2013; Pei et al., 2018; Wu et al., 2020). Alternatively, genome-wide association studies (GWAS) based on the use of natural population, in contrast to linkage analysis using bi-parental populations, have more extensive recombination events and, thus, result in less short LD segments leading to increased resolution and accuracy of marker-phenotype associations (Duan et al., 2022; Liang et al., 2022). In this study, hundreds of SNPs loci were found to be significantly associated with the individual and TIF content, and they were distributed across all 20 chromosomes of soybean. Furthermore, many of these SNPs were simultaneously identified in five environments, as observed in malonylglycitin, malonylgenistin, and four environments like total isoflavones, malonyldaidzin, malonylgenistin, malonylglycitin, etc. Most of the significantly associated SNPs were observed for individual and total isoflavones, underlying that a high portion of the *G. max* genome has genomic regions harboring many candidate SNPs based on the wide diverse panel of soybean accessions utilized in the current study. These findings are consistent with a previous study (Wu et al., 2020) that found significant loci for both individual and TIF content across several sites in a natural soybean population.

WGCNA analysis is an effective technique for categorizing the transcriptome data into co-expression modules to reduce the number of potential candidate genes (Hollender et al., 2014; Greenham et al., 2017; Schaefer et al., 2018; Azam et al., 2023). In this study, out of eight modules, three modules were positively associated with individual and TIF content. The expression patterns of genes present in these modules revealed a higher expression at the late seed development stage. Previous studies also reported that the accumulation of isoflavones mainly occurs at the late stage of seed development (Jung et al., 2000; Dhaubhadel et al., 2003; Cheng et al., 2008; Azam et al., 2023). In addition, GO analysis of these modules revealed some significant GO terms related to biotic and abiotic stresses. Devi et al. (2020) reported that biotic and abiotic stresses lead to an increase isoflavone accumulation by the upregulation of *IFS1* and *IFS2* genes at the late seed development stage. While Uchida et al. (2020) also found that isoflavone O-methyltransferase (*GmIOMT1*) produced higher levels of glycitein in response to biotic stress. Therefore, identifying genes involved in these modules would provide new genetic resources to better understand the isoflavone biosynthesis pathway.

We have identified 27 key candidate genes from brown, magenta, and green modules. Brown module, which showed the highest correlation and gene significance with TIF, contained a cytochrome P450 (*Glyma.08G125100*). A branch of the phenylpropanoid pathway synthesizes isoflavones. Cytochrome P450 play a crucial role in the biosynthesis of a wide variety of plant metabolites (Chapple, 1998). Isoflavone synthases (*IFS1* and *IFS2*) are the members of cytochrome P450 super gene family and play a vital role in isoflavone accumulation by producing the 2-hydroxyisoflavone by catalyzing the flavone intermediates (naringenin and liquiritigenin) (Liu et al., 2002). The MYB transcription factors play crucial roles in the regulation of isoflavone biosynthesis by triggering the gene expression of key isoflavonoid biosynthesis enzymes, namely, chalcone isomerases (*CHI*), chalcone synthases (*CHS*), isoflavone synthases (*IFS1* and *IFS2*) (Yi et al., 2010; Chu et al., 2017). We identified *MYB133* as a key candidate gene which was previously identified by (Bian et al., 2018) as a positive regulator of isoflavones through genome-wide analysis, which directly activates *IFS2* and CHS8 and promotes isoflavone accumulation. We identified the natural variation of *MYB133* in the natural population of soybean, which showed a higher TIF level across different regions, landraces, and cultivars (**Supplementary Figure 4**). Furthermore, the natural variation in the bZIP transcription factor caused synonymous mutation which revealed significant variations for individual and total isoflavones. Previous studies also reported that the synonymous mutations are not just silent but also cause a significant change in the phenotypes (Chu and Wei, 2020; Shen et al., 2022). The bZIP transcription factors are previously reported to control isoflavone accumulation by interacting with MYB transcription factors and play an important role against biotic and abiotic stresses in soybean (He et al., 2020; Yang et al., 2020; Anguraj Vadivel et al., 2021). In addition to MYB and bZIP transcription factors, different zinc-finger transcription factors, such as *GmZFP7*, *GmVOZs*, and *GsVOZs*, regulate isoflavone and stress responses in soybean. (Rehman et al., 2021; Feng et al., 2023)

These findings suggest that most identified key candidate genes include enzymes and transcription factors from important gene families involved in isoflavone biosynthesis. So, the functional validation of these key candidate genes will provide new insights to better understand the molecular mechanism underlying isoflavone biosynthesis.

## 5 Conclusion

The current study demonstrated that GWAS analysis using natural populations is an effective strategy for identifying candidate genes in soybean. Based on the GWAS and WGCNA, 3 modules were identified that were highly correlated with individual and TIF content. Within these modules, we have identified four key candidate genes and the natural variation present in *Glyma.11G108100* revealed that it influences the isoflavone accumulation in soybean seed. The functional analysis of *Glyma.11G108100* will provide new insight to better understand the isoflavone synthesis pathway.

## Data availability statement

The original contributions presented in the study are publicly available. This data can be found here: https://sfgb.rmbreeding.cn/search/gemplasm, 16NF1005_1006, corresponding accession name Dongnong4hao, ID number ZDD00023.

## Author contributions

MAz, Investigation, data curation, visualization, writing-original draft preparation, SZ, LJ, supervision, conceptualization, methodology, investigation, data curation, MAh, KGAB, JQ, resources, formal analysis, software, YF, YL, LQ and BL resources, project administration, conceptualization, writing-review, and editing, JS, funding acquisition, supervision, conceptualization, visualization, writing-review, and editing. All authors contributed to the article and approved the submitted version.
