# Supplementary material for: Identification of hub genes regulating isoflavone accumulation in soybean seeds via GWAS and WGCNA approaches
Source: Front Plant Sci. 2023 Feb 14;14:1120498. doi: 10.3389/fpls.2023.1120498 (PMC9971994; doi:10.3389/fpls.2023.1120498)
Supplement: Supplementary file 1 [file DataSheet_1.docx]

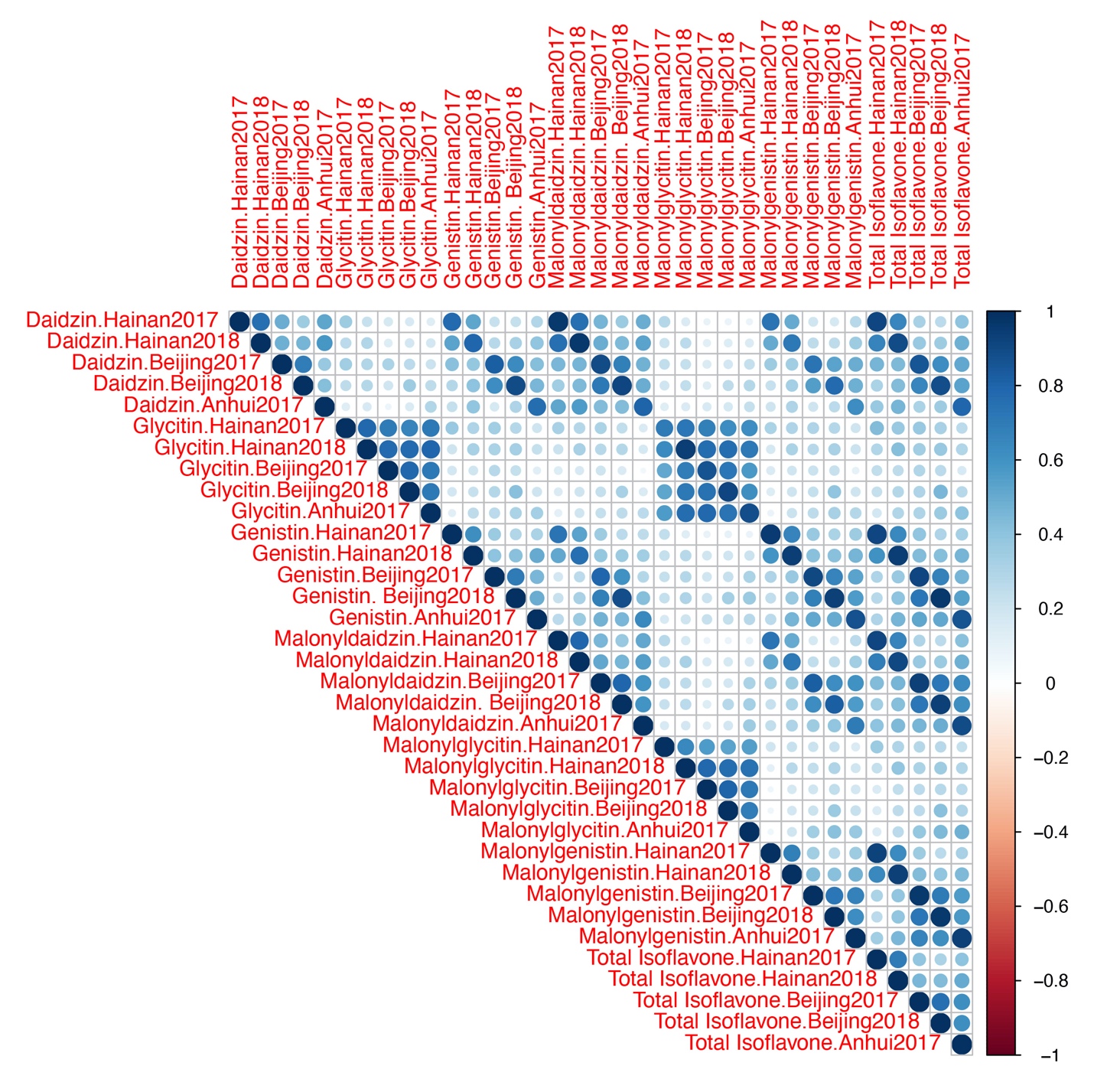


**Supplementary Figure 1.** The correlations among the five environments for individual and total isoflavone content.


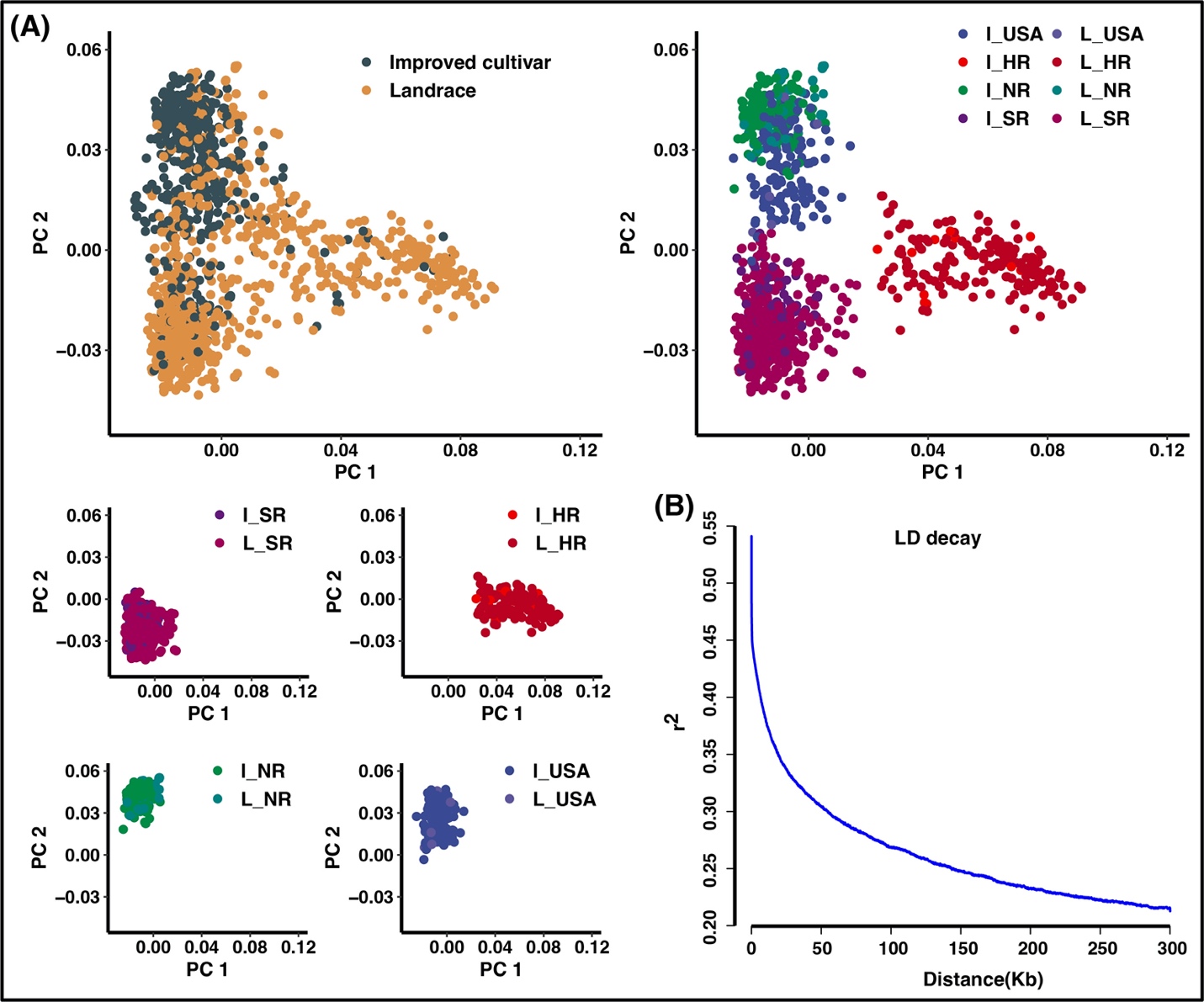


**Supplementary Figure 2. (A)** Genetic structure **(B)** Linkage disequilibrium of 1551 soybean accessions (I_, Improved cultivar; L_, Landrace; NR, Northern region; HR, Huang Huai Hai valley region; SR, Southern region; USA, United States of America)

**
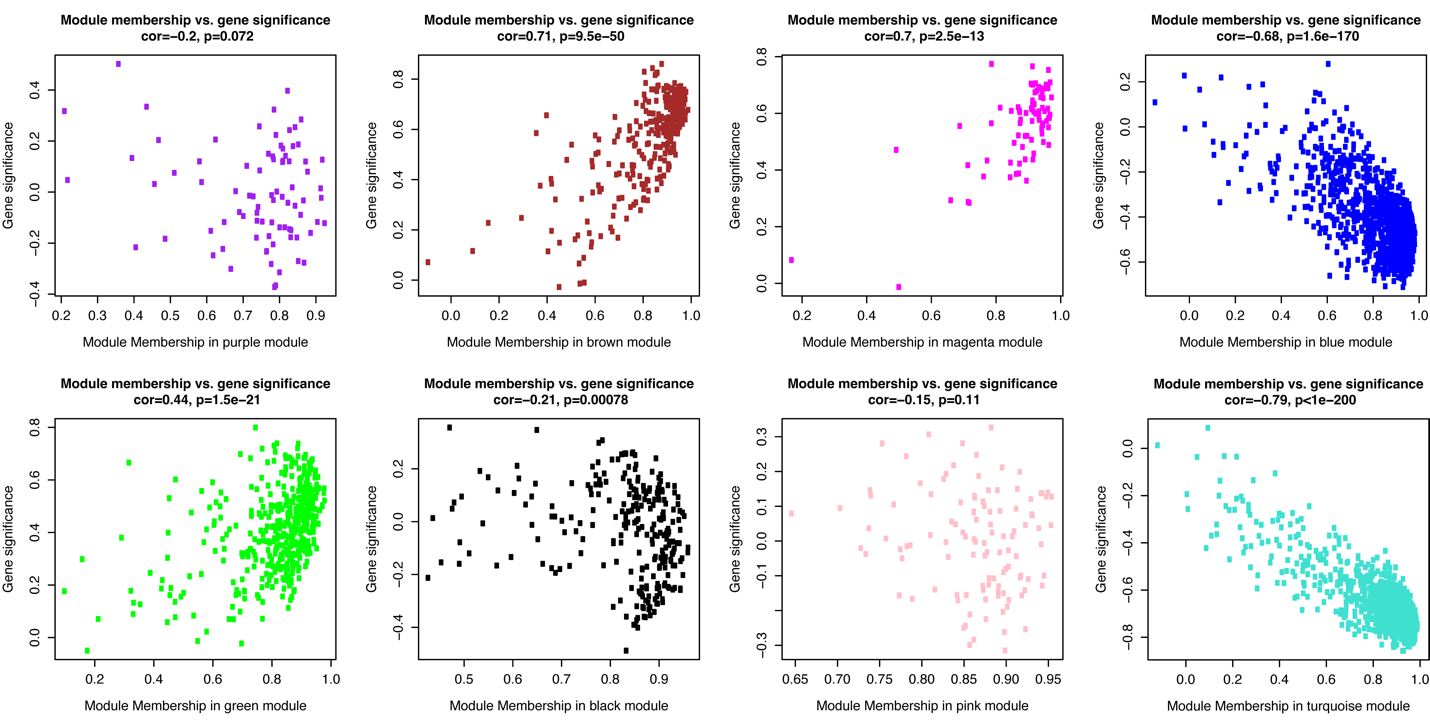
**

**Supplementary Figure 3.** TIF gene significance VS module membership


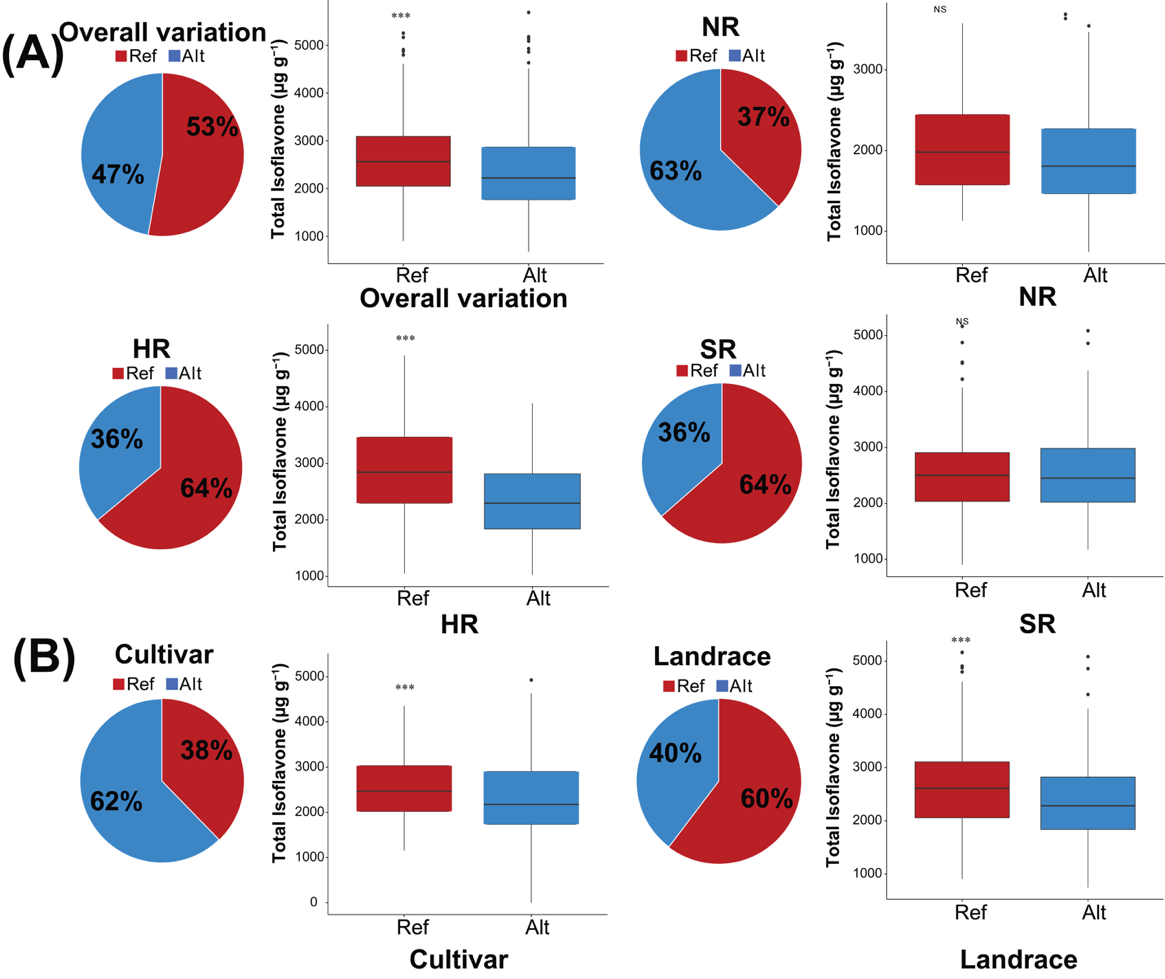


**Supplementary Figure 4. (A)** Natural variation of *Glyma.07G066100* for TIF content **(B)** Natural variation of *Glyma.07G066100* for cultivar and landraces.
